# Ciliary body suturing using intraocular irrigation for traumatic cyclodialysis: two case reports

**DOI:** 10.1186/s13256-020-02448-z

**Published:** 2020-08-06

**Authors:** Taishi Nagashima, Ryohsuke Kohmoto, Masanori Fukumoto, Shou Oosuka, Takaki Sato, Takatoshi Kobayashi, Teruyo Kida, Tsunehiko Ikeda

**Affiliations:** Department of Ophthalmology, Osaka Medical College, 2-7 Daigaku-machi, Takatsuki City, Osaka, 569-8686 Japan

**Keywords:** Cyclodialysis, Ciliary body suturing, Intraocular irrigation

## Abstract

**Background:**

We report two cases (two eyes) of traumatic cyclodialysis with prolonged decrease of intraocular pressure that were successfully treated with surgery by ciliary body suturing using intraocular irrigation.

**Case presentation:**

This study involved a 17-year-old Japanese boy (patient 1) and a 31-year-old Japanese man (patient 2) in whom cyclodialysis was sustained in one eye after blunt-force ocular trauma from a thrown ball. Because the hypotony maculopathy in both patients did not resolve after conservative treatment, ciliary body suturing was performed. Briefly, a 25-gauge trocar intraocular irrigation needle for vitrectomy was inserted into the vitreous cavity of the injured eye. A lamellar scleral flap was then made, and an incision was created on the sclera while maintaining intraocular pressure. Next, the detached ciliary body was sutured to the sclera under direct vision. The intraocular fluid at the site of cyclodialysis was then rapidly drained from the scleral wound because of elevated intraocular pressure in the vitreous cavity, and the ciliary body was visually recognized through the scleral wound under direct vision, thus allowing a stable suture fixation of the ciliary body to the sclera. Postoperatively, the treated eye in both cases showed improvement of intraocular pressure and visual function.

**Conclusions:**

The surgical method described in this report was found to be effective for draining intraocular fluid at the site of cyclodialysis and for performing a stable suture fixation of the ciliary body to the sclera through the scleral wound under direct vision, and it should be considered advantageous for avoiding intraoperative bleeding and suturing in a blinded manner.

## Background

The current surgical treatments for cyclodialysis include ciliary body suturing [1–3], cyclocryotherapy [4–6], laser photocoagulation [7–10], encircling [11, 12], intraocular lens (IOL) suture fixation [13–16], and vitreous surgery [17–21]. Although there are numerous surgical procedures available, each has its own specific advantages and disadvantages. We report two cases of blunt force trauma–related cyclodialysis and resultant hypotony maculopathy that were successfully treated via suturing of the ciliary body to the sclera with a 25-gauge trocar for vitrectomy using intraocular irrigation under direct vision.

## Case presentation

### Patient 1

**Fig. 1:**
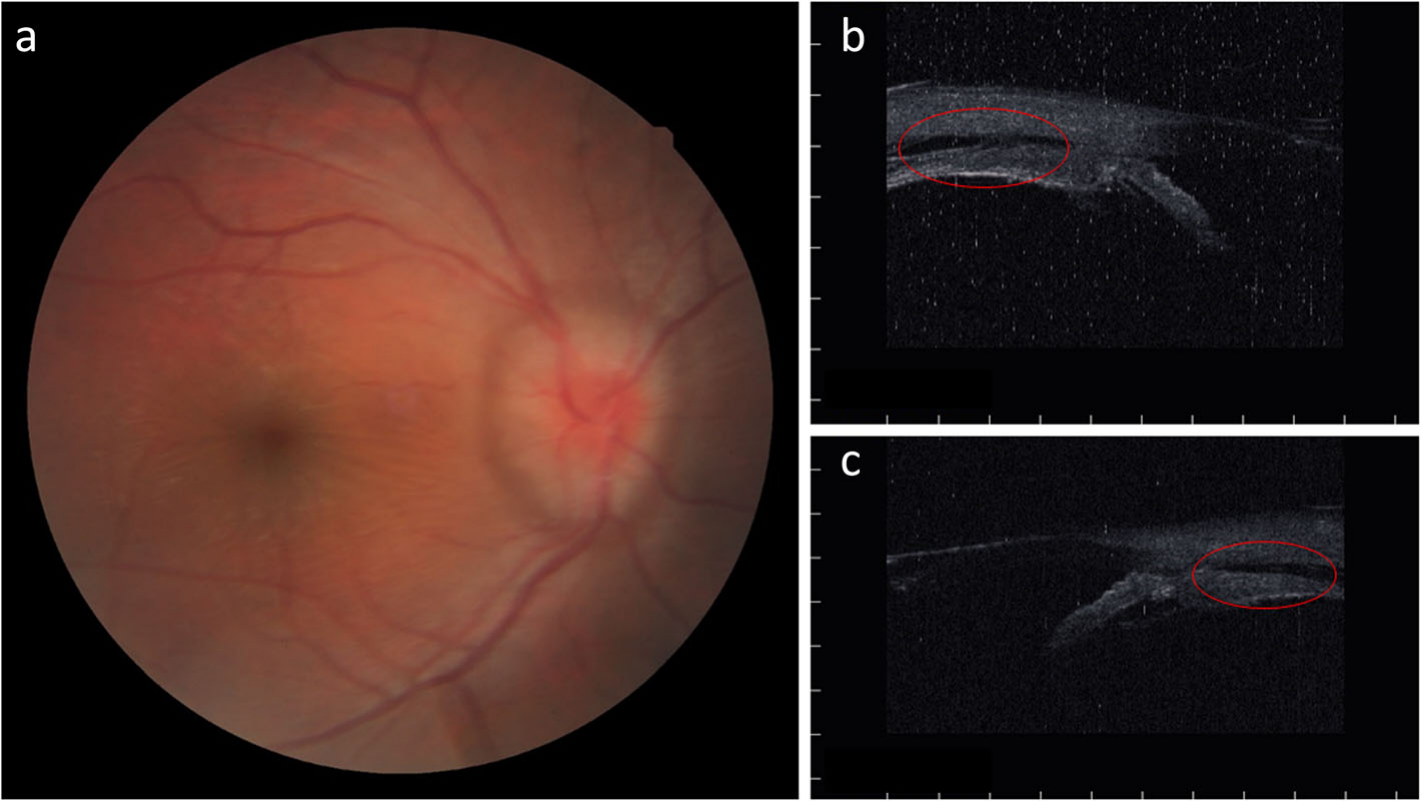
Preoperative fundoscopy and ultrasound biomicroscopy (UBM) images in patient 1. **a** Fundoscopic image showing macular fold, papilledema, and retinal vessel dilatation. **b** and **c** UBM images showing cyclodialysis from the upper region to the temporal region and in the lower areas

**Fig. 2:**
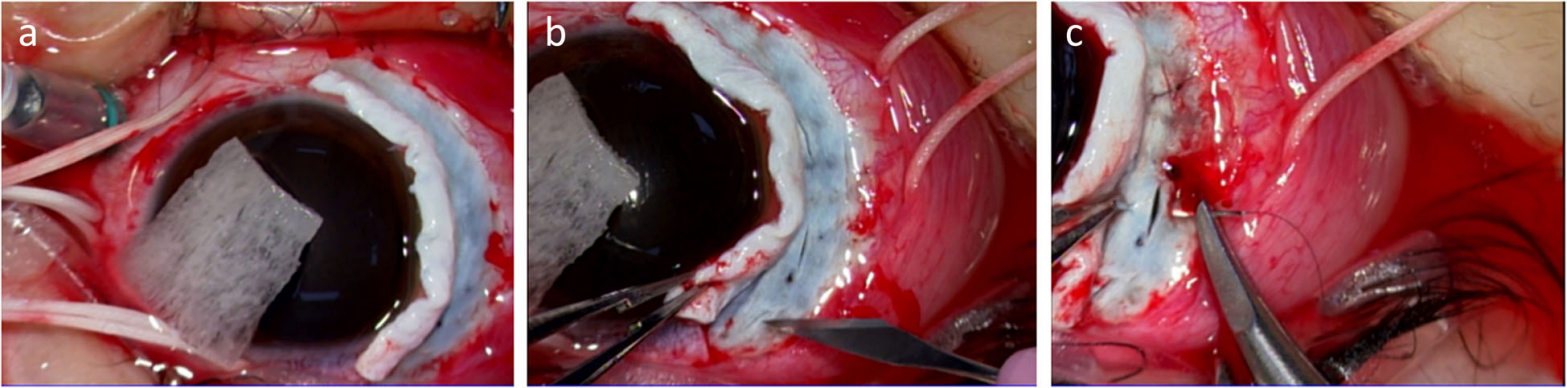
Intraoperative findings in patient 1. After preparing a lamellar scleral flap in the 6- and 12-o’clock directions (**a**), three incisions were made on the sclera under the scleral flap, and the intraocular fluid at the site of the cyclodialysis was easily drained (**b**). The ciliary body was fixed to the sclera using a 10-0 nylon suture under direct vision (**c**)

**Fig. 3:**
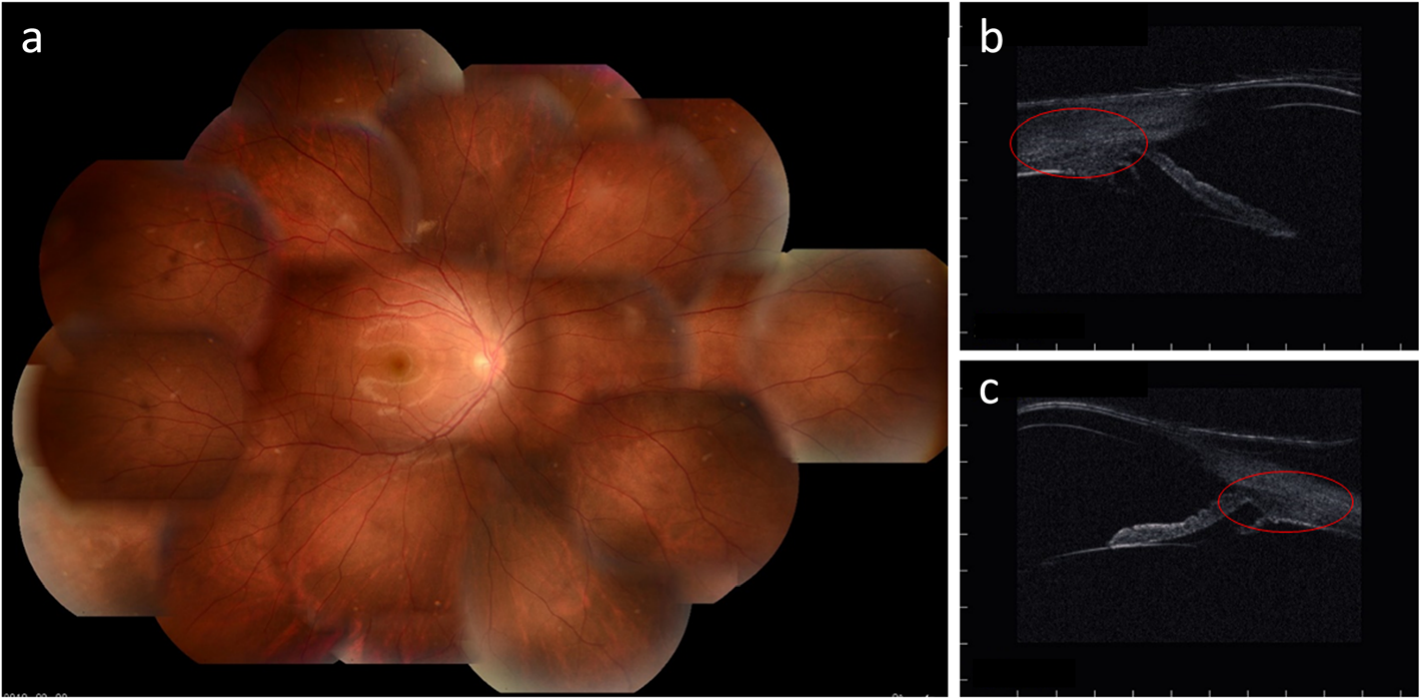
Postoperative fundoscopy and ultrasound biomicroscopy (UBM) images in patient 1. **a** Fundoscopic image showing improvement after surgery. **b** and **c** UBM images showing that the cyclodialysis had resolved

An 17-year-old Japanese boy presented with a periorbital hematoma and the development of an anterior chamber hemorrhage in his right eye that had developed after he was hit with a softball 2 months earlier. Thereafter, the anterior chamber hemorrhage resolved; yet, decreased vision and decreased intraocular pressure (IOP) persisted. His past medical history was unremarkable, and fundoscopic examination and optical coherence tomography (OCT) revealed hypotony maculopathy. Ultrasound biomicroscopy (UBM) of the anterior segment (AS) revealed cyclodialysis, which was surgically treated at 5 months after the patient’s injury. Prior to surgery, the visual acuity (VA) and IOP in the patient’s right eye was 0.04 (0.15 × S-4.50 C − 3.50 Ax 5 degrees) and 4 mmHg, respectively. AS-OCT examination revealed a shallow anterior chamber, a macular fold in the fundus, papilledema, and retinal vessel dilation, and UBM imaging revealed cyclodialysis from the upper to temporal regions and in the lower areas (Fig. 1a–c). For treatment, a 25-gauge trocar intraocular irrigation needle was inserted into the lower nasal side of the right eye. Next, and after confirming that the tip of the needle was correctly inserted into the vitreous cavity, irrigation fluid was injected into the eye at the pressure of 20 mmHg. After preparing a lamellar scleral flap in the 6- and 12-o’clock directions, three incisions were made on the sclera under the scleral flap, and the intraocular fluid at the site of the cyclodialysis was easily drained due to the elevated pressure at the intraocular irrigation port, by which the ciliary body was visually recognized through the scleral wound under direct vision. Next, diathermy coagulation was performed under the scleral flap, and the ciliary body was securely fixed to the sclera using a 10-0 nylon suture under direct vision (Fig. 2a–c). Intraoperatively, there was no bleeding from the suture site, and the lamellar scleral flap was then closed to complete the surgery. After surgery, the VA and IOP in his right eye improved to (1.0 × S -3.00 = C − 1.25 Ax 180 degrees) and 13 mmHg, respectively, with good visual function. Moreover, UBM examination revealed that the cyclodialysis had resolved and that the initial fundoscopy findings had improved (Fig. 3a–c).

### Patient 2

**Fig. 4:**
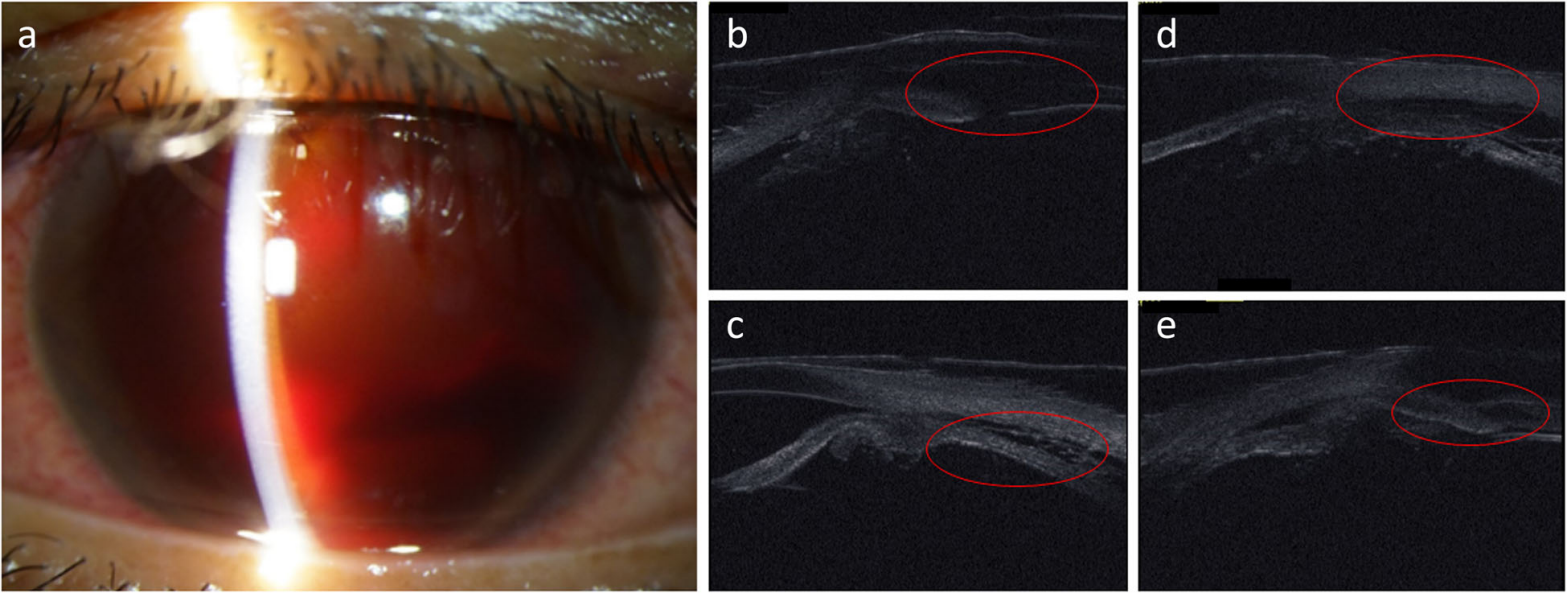
Preoperative slit-lamp and ultrasound biomicroscopy (UBM) images in patient 2. **a** Fundoscopic image showing anterior chamber hemorrhage, traumatic mydriasis, traumatic cataract, and phacodonesis. **b–e** UBM images showing circumferential cyclodialysis

**Fig. 5:**
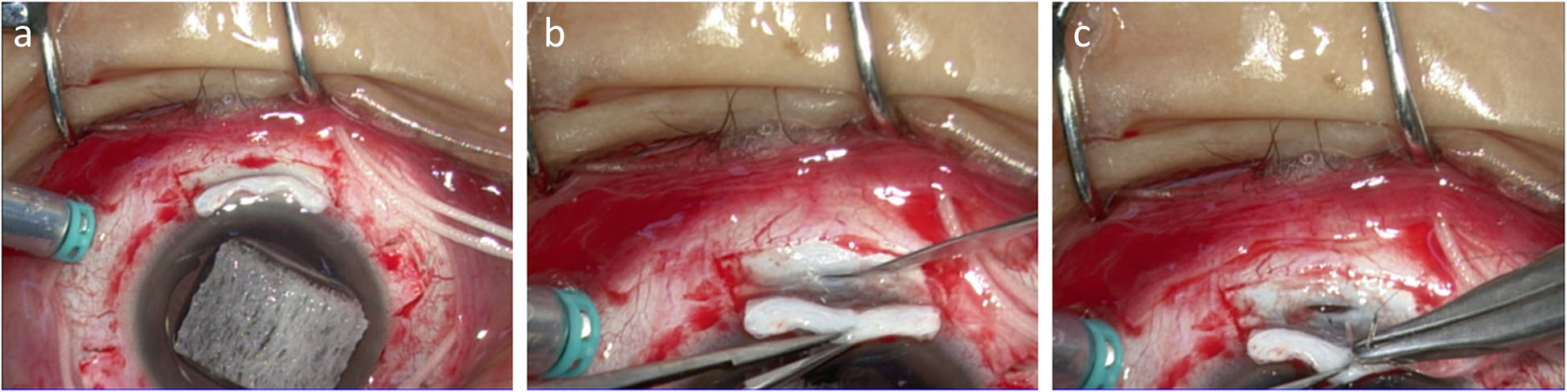
Intraoperative findings in patient 2. Images showing the lamellar scleral flap made in the lower and upper parts (**a**) and the incision made on the sclera (**b**). As in patient 1, the intraocular fluid at the site of the cyclodialysis was easily drained through the incision site, and the advancing ciliary body was fixed to the sclera using a 10-0 nylon suture under direct vision (**c**)

**Fig. 6:**
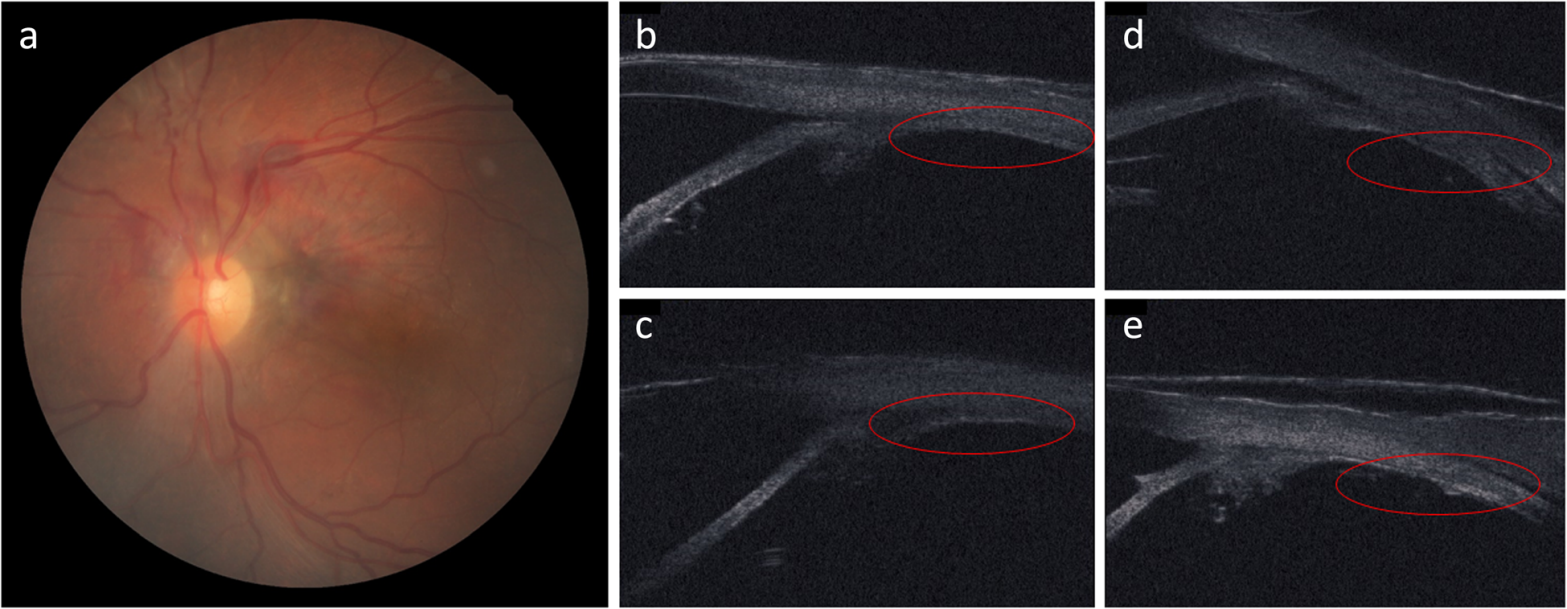
Postoperative fundoscopy and ultrasound biomicroscopy (UBM) images in patient 2. **a** Fundoscopic image showing improvement after surgery. **b–e** UBM images showing that the cyclodialysis had nearly resolved

A 32-year-old Japanese man presented to our hospital with a periorbital hematoma and the development of an anterior chamber hemorrhage in his left eye due to being hit with a rubber ball. His past medical history was unremarkable, and upon initial examination, the VA and IOP in his right eye were 30 cm/vision (i.e., counting fingers; noncorrigent uncorrected) and 4 mmHg, respectively. AS-OCT examination revealed subconjunctival hemorrhage, corneal stromal and epithelial edema with Descemet membrane folds, hemorrhage in the anterior chamber, traumatic mydriasis and cataract, phacodonesis, and a mild vitreous hemorrhage. Although ultrasound B-mode imaging showed no retinal detachment, UBM imaging revealed circumferential cyclodialysis (Fig. 4a–e). Moreover, although the anterior chamber hemorrhage and vitreous hemorrhage gradually disappeared, there was a persistent decreased of IOP. At 1 month after injury, the patient underwent vitrectomy, lensectomy, IOL transscleral sulcus fixation, and ciliary body suturing in his left eye. Briefly, a 25-gauge trocar intraocular irrigation needle was inserted, and after confirmation that the tip of the needle was correctly inserted into the vitreous cavity, irrigation fluid was injected into the eye at the pressure of 20 mmHg. Next, pars plana lensectomy and vitrectomy were performed, and fundoscopic examination showed hypotony maculopathy. Thus, transscleral sulcus fixation of the IOL was then performed. In addition, the pupil was adjusted by suture tightening using a 10-0 PROLENE suture (Ethicon/J&J Medical Devices, Bridgewater, NJ, USA) in the upper and lower parts for the treatment of traumatic mydriasis. A lamellar scleral flap was then made in the lower and upper regions, and an incision was made on the sclera. As in patient 1, the intraocular fluid at the site of cyclodialysis was drained from the incision site, and, when within range, the ciliary body was fixed to the sclera using 10-0 nylon suture under direct vision (Fig. 5a–c). Intraoperatively, there was no bleeding from the suture site, and at 2 months after surgery, the VA and IOP in his left eye had improved to (0.6 × S + 2.75 = C-2.00 Ax 50 degrees) and 12 mmHg, respectively, with good visual function. Moreover, UBM imaging revealed that the cyclodialysis had nearly resolved and that the initial fundoscopic findings had improved (Fig. 6a–e).

## Discussion

Cyclodialysis is a disease that usually occurs due to blunt force trauma. In such cases, the ciliary body is separated from the sclera, which reduces aqueous production and allows the flow of aqueous humor through the cleft into the suprachoroidal space, thus leading to a reduction of IOP. Cyclodialysis can cause hypotony maculopathy (including a macular fold), dilated and tortuous retinal vessels, and papilledema, and it can also lead to irreversible vision loss if allowed to persist over a long-term period [1]. Although conservative treatment alone can sometimes result in improvement of the decreased IOP and the hypotony maculopathy can spontaneously resolve, surgical treatment is required for patients with long-lasting symptoms.

As described above, the current surgical treatments for cyclodialysis include ciliary body suturing [1–3], cyclocryotherapy [4–6, 22], laser photocoagulation [7–10], encircling [11, 12], IOL suture fixation [13–16], and vitreous surgery [17–21]. Cyclocryotherapy and laser photocoagulation are performed to close the cyclodialysis cleft via the creation of an adhesion between the ciliary body and the sclera. However, because the sclera is the tissue that contains less pigment, the adhesion created by the coagulation procedure may be unsatisfactory. Thus, suturing of the ciliary body is generally considered to be a more effective method of obtaining a secure adhesion, even though the procedure is invasive and can possibly result in bleeding from the ciliary body due to the suturing being performed in a blinded manner.

In the surgical method used for the two cases described in this report, a trocar tip for vitrectomy is inserted into the vitreous cavity in order to maintain the intraoperative IOP, and this procedure provided the following three advantages. First, the intraocular fluid at the site of the cyclodialysis was easily drained from the scleral wound due to the IOP being well maintained during the scleral incision. Second, because the separated ciliary body advancing toward the sclera could be visually observed under direct vision, it was possible to perform a stable 10-0 nylon suturing of the ciliary body to the advancing sclera. Third, because the IOP was well maintained intraoperatively, the risk of bleeding from the ciliary body during suturing was reduced.

However, it should be noted that the surgical procedure does have some disadvantages. Those include a slightly longer time being required to create the lamellar scleral flap, as well as the incarceration of the ciliary body into the scleral wound that occurs when the intraocular irrigation pressure is set at a higher level or when the scleral incisional wound is large. Moreover, the insertion of the 25-gauge trocar tip into the vitreous cavity may be difficult in cases of severe cyclodialysis. However, in such cases, tilting of the eyeball can resolve the problem because it helps to confirm the position of the transpupillary tip. For optimal results, we consider that the specific site of the trocar placement should be selected from the area in which the preoperative UBM findings indicate that the cyclodialysis is mild.

Previous studies have reported the effectiveness of other surgical methods for the treatment of cyclodialysis, such as compression of the detached ciliary body with a loop of the IOL or capsular tension ring, and IOL ciliary sulcus suture fixation, a method involving direct suturing of the ciliary body to the sclera [13–16]. In patient 2, the combined use of IOL ciliary sulcus suture fixation allowed us to fix the detached ciliary body to the sclera in the nasal and temporal sides, thus allowing us to create a lamellar scleral flap only in the upper and lower parts. In the future, we theorize that a combination of these surgical procedures will improve the outcomes of ciliary body suturing.

## Conclusions

The surgical method used in the present study was found to be effective for draining the intraocular fluid at the site of the cyclodialysis and for performing a stable suture fixation of the ciliary body to the sclera through the scleral wound under direct vision.

## Abbreviations

AS: Anterior segment
IOL: Intraocular lens
IOP: Intraocular pressure
OCT: Optical coherence tomography
UBM: Ultrasound biomicroscopy
VA: Visual acuity
